# Enhanced Bioconversion of Cellobiose by Industrial *Saccharomyces cerevisiae* Used for Cellulose Utilization

**DOI:** 10.3389/fmicb.2016.00241

**Published:** 2016-03-03

**Authors:** Meng-Long Hu, Jian Zha, Lin-Wei He, Ya-Jin Lv, Ming-Hua Shen, Cheng Zhong, Bing-Zhi Li, Ying-Jin Yuan

**Affiliations:** ^1^Key Laboratory of Systems Bioengineering (Ministry of Education), School of Chemical Engineering and Technology, Tianjin UniversityTianjin, China; ^2^Collaborative Innovation Center of Chemical Science and Engineering (Tianjin), Tianjin UniversityTianjin, China; ^3^Key Laboratory of Industrial Fermentation Microbiology (Ministry of Education), Tianjin University of Science and TechnologyTianjin, China

**Keywords:** synthetic biology, cellobiose utilization, industrial strain, evolution engineering, simultaneous saccharification and fermentation

## Abstract

Cellobiose accumulation and the compromised temperature for yeast fermentation are the main limiting factors of enzymatic hydrolysis process during simultaneous saccharification and fermentation (SSF). In this study, genes encoding cellobiose transporter and β-glucosidase were introduced into an industrial *Saccharomyces cerevisiae* strain, and evolution engineering was carried out to improve the cellobiose utilization of the engineered yeast strain. The evolved strain exhibited significantly higher cellobiose consumption rate (2.8-fold) and ethanol productivity (4.9-fold) compared with its parent strain. Besides, the evolved strain showed a high cellobiose consumption rate of 3.67 g/L/h at 34°C and 3.04 g/L/h at 38°C. Moreover, little cellobiose was accumulated during SSF of Avicel using the evolved strain at 38°C, and the ethanol yield from Avicel increased by 23% from 0.34 to 0.42 g ethanol/g cellulose. Overexpression of the genes encoding cellobiose transporter and β-glucosidase accelerated cellobiose utilization, and the improvement depended on the strain background. The results proved that fast cellobiose utilization enhanced ethanol production by reducing cellobiose accumulation during SSF at high temperature.

## Introduction

Cellulosic ethanol is widely recognized as a potential alternative fuel for its renewable, environmental and strategic attributes (Zhong et al., 2010). Simultaneous saccharification and fermentation (SSF) is one of the important processes for cellulosic ethanol production (Hahn-Hägerdal et al., 2006; Zhu et al., 2015). Combining the consumption and release of glucose, SSF improves the efficiency of enzymatic hydrolysis compared with separate hydrolysis and fermentation (SHF; Tomás-Pejó et al., 2008). The entire SSF process is carried out in a single reactor, thereby curbs the equipment costs and saves energy (Brethauer and Wyman, 2010). Despite these advantages, the cellobiose accumulation and the different optimal temperatures of cellulolytic enzymes (around 50°C) and yeast fermentation (around 30°C) are the key issues to further improve SSF efficiency (Olofsson et al., 2008; Zhu et al., 2014).

In SSF the traditional saccharification process relies on the addition of a cellulase cocktail consisting of endoglucanases (EG), cellobiohydrolases (CBH), and β-glucosidases (BGL; Li B. Z. et al., 2010; Zhang et al., 2012). During the process, cellulose is firstly degraded into glucose by the synergistic action of three classes of enzymes and then the glucose is converted into ethanol by the fermentation strains (Horn et al., 2012). However, fungal cellulases exhibit weak β-glucosidase (BGL) activity which results in slow degradation of cellobiose into glucose, thus cellobiose is accumulated during SSF (Chauve et al., 2010). Since cellobiose is one of the significant inhibitors of cellulases (Andrić et al., 2010; Hsieh et al., 2014), the overall rate and efficiency of enzymatic hydrolysis of cellulose is inhibited by the accumulation of cellobiose (Singhania et al., 2013; Qin et al., 2014). To increase the efficiency of enzymatic hydrolysis, extra BGL needs to be added in during the enzymatic hydrolysis, which results in a higher cost (Zhang et al., 2010). In order to reduce the cost of enzymes in ethanol production, microorganisms capable of utilizing cellobiose have been developed (la Grange et al., 2010). *Saccharomyces cerevisiae* is preferred in the industrial ethanol production due to its robustness, industrial-scale and high ethanol productivity (Nielsen et al., 2013). Since native *S. cerevisiae* cannot ferment cellobiose, three strategies have been developed for heterologous cellobiose utilization. In the first strategy, *S. cerevisiae* was engineered to secrete or display a BGL to hydrolyze cellobiose to glucose outside the cells (Matsuoka et al., 2014; Treebupachatsakul et al., 2016). The phosphorolytic pathway is the second strategy which involves heterologous expression of a cellobiose phosphorylase and a lactose permease or a cellobiose transporter (CDT). The cellobiose is transported into the cell via the lactose permease or CDT and then cleaved by the cellobiose phosphorylase in the presence of inorganic phosphate to produce glucose and α-glucose-1-phosphate (Sadie et al., 2011; Ha et al., 2013a; Chomvong et al., 2014). The third strategy is the hydrolytic pathway. Heterologous cellobiose hydrolytic pathway consisting of a CDT and a BGL was introduced into a laboratory *S. cerevisiae* strain (Galazka et al., 2010). In the engineered yeast strain, cellobiose is transported into the cell by the CDT and hydrolyzed to glucose by the BGL and then metabolized by the cell. However, the ethanol yield and cellobiose fermentative efficiency are not high enough when using engineered yeast strains with heterologous pathways, and further engineering is required to improve the cellobiose utilization.

Another concern of SSF is the mismatch between the optimal temperatures for enzymatic hydrolysis (about 50°C) and yeast fermentation (about 30°C; Weber et al., 2010). To reach a compromise between the optimal temperatures for enzymes and yeast cells, SSF is commonly performed at lower temperature which leads to a poor hydrolysis efficiency of the substrate (Hasunuma and Kondo, 2012). Therefore, thermotolerant microbial strains capable of producing ethanol at higher temperatures are essential for the improvement of SSF efficiency (Lin and Tanaka, 2006). Thermotolerant yeast strains, including *Kluyveromyces fragilis* (Hari Krishna et al., 2001), *Pichia kudriavzevii* (Koutinas et al., 2016), *Hansenula polymorpha* (Kurylenko et al., 2014), *Kluyveromyces marxianus* (Arora et al., 2015; Wu et al., 2016) and other newly isolated *Kluyveromyces* sp. (Narra et al., 2015) have been used in SSF at temperatures close to the optimal temperature for enzymatic hydrolysis. Many studies have focused on *S. cerevisiae* strains due to the robustness for ethanol production, and thermotolerant strains were obtained and applied in SSF (Park et al., 2010; Chu et al., 2012; Cha et al., 2015). In order to further improve the enzymatic hydrolysis efficiency during SSF, thermotolerant *S. cerevisiae* capable of rapid cellobiose utilization is required.

In this study, cellobiose utilization pathway was constructed in a thermotolerant industrial *S. cerevisiae* strain. An evolved strain SyBE001603 was obtained through evolution engineering and characterized at different temperatures (30–42°C). The efficiency of SSF was significantly improved at high temperature by using strain SyBE001603 with fast cellobiose utilization.

## Materials and methods

### Strains and culture media

*Escherichia coli* DH5α was used as the host strain for gene cloning and manipulation. The *S. cerevisiae* strain L2612 used as the host for DNA assembly was a gift from Professor Thomas Jeffries at University of Wisconsin–Madison (Zha et al., 2013). The industrial *S. cerevisiae* strain SyBE001601 used as chassis in this study was a commercial strain and reported in our previous study (Ding et al., 2012). Yeast strains and plasmids used in this work are described in Table 1.

**Table 1:** **Strains and plasmids used in this study**.

**Strains and plasmids**	**Description**	**Source or references**
**PLASMIDS**		
pRS426-*cdt-1*	P_PGK1_-*cdt-1*-T_CYC1_	Galazka et al., 2010
pRS425-*gh1-1*	P_PGK1_-*gh1-1*-T_CYC1_	Galazka et al., 2010
pRS426-*cdt-1-gh1-1*	P_PGK1_-*cdt-1*-T_CYC1_-P_PGK1_-*gh1-1*-T_CYC1_	This study
pRS415		Brachmann et al., 1998
pRS415-*cdt-1*	P_PGK1_-*cdt-1*-T_CYC1_	This study
pRS415-*gh1-1*	P_PGK1_-*gh1-1*-T_CYC1_	This study
**STRAINS**		
SyBE001601	Angel Yeast, Product No. 80000012	Ding et al., 2012
SyBE001602	SyBE001601, pRS426-*cdt-1-gh1-1*	This study
SyBE001603	Evolved from SyBE001602	This study
SyBE001604	SyBE001601, isolated plasmid from SyBE001603	This study
L2612	*MATα leu2-3 leu2-112 ura3-52trp1-298 can1 cyn1 gal+*	Zha et al., 2013
L2612-Cell	L2612/pRS426-*cdt-1-gh1-1*	This study
L2612-Cell+CDT	L2612/pRS426-*cdt-1-gh1-1*/pRS415-*cdt-1*	This study
L2612-Cell+BGL	L2612/pRS426-*cdt-1-gh1-1*/pRS415-*gh1-1*	This study
BY4741	*MATa his3Δ1 leu2Δ0 met15Δ0 ura3Δ0*	Brachmann et al., 1998
BY4741-Cell	BY4741/pRS426-*cdt-1-gh1-1*	This study
BY4741-Cell+CDT	BY4741/pRS426-*cdt-1-gh1-1*/pRS415-*cdt-1*	This study
BY4741-Cell+BGL	BY4741/pRS426-*cdt-1-gh1-1*/pRS415-*gh1-1*	This study
BY4742	*MAT*α *his3*Δ1 *leu2*Δ0 *lys2*Δ0 *ura3*Δ0	Brachmann et al., 1998
BY4742-Cell	BY4742/pRS426-*cdt-1-gh1-1*	This study
BY4742-Cell+CDT	BY4742/pRS426-*cdt-1-gh1-1*/pRS415-*cdt-1*	This study
BY4742-Cell+BGL	BY4742/pRS426-*cdt-1-gh1-1*/pRS415-*gh1-1*	This study
YSG50	*MAT*α *ade2–1 ade3*Δ*22 ura3–1 his3–11 15 trp1–1 leu2–3 112 and can1–100*	Shao et al., 2009
YSG50-Cell	YSG50/pRS426-*cdt-1-gh1-1*	This study
YSG50-Cell+CDT	YSG50/pRS426-*cdt-1-gh1-1*/pRS415-*cdt-1*	This study
YSG50-Cell+BGL	YSG50/pRS426-*cdt-1-gh1-1*/pRS415-*gh1-1*	This study
CEN.PK2-1C	*MATa ura3-52 trp1-289 leu2-3_112 his3Δ1 MAL2-8 c SUC2*	From EUROSCARF
CEN.PK2-1C-Cell	CEN.PK2-1C/pRS426-*cdt-1-gh1-1*	This study
CEN.PK2-1C-Cell+CDT	CEN.PK2-1C/pRS426-*cdt-1-gh1-1*/pRS415-*cdt-1*	This study
CEN.PK2-1C-Cell+BGL	CEN.PK2-1C/pRS426-*cdt-1-gh1-1*/pRS415-*gh1-1*	This study

*E. coli* was cultured in Luria-Bertani medium (5 g/L yeast extract, 10 g/L tryptone, 10 g/L NaCl) at 37°C and 250 rpm, and 100 mg/mL of ampicillin was added to the medium when necessary. Yeast strains were cultivated in YP medium (10 g/L yeast extract, 20 g/L peptone) supplemented with 20 g/L glucose (YPD) or 20 g/L cellobiose (YPC), at 30°C and 200 rpm (for aerobic growth) or 150 rpm (for oxygen limited conditions). SC agar medium containing 6.7 g/L yeast nitrogen base, 2 g/L appropriate nucleotides and amino acids, 20 g/L cellobiose and 20 g/L agar was used for selection of transformants.

### Plasmid and strain construction

The plasmids were constructed as follows. The plasmid pRS426-*cdt-1* was linearized by restriction enzyme NgoMIV. The expression cassette of BGL gene with the *PGK1* promoter and the *CYC1* terminator was amplified by PCR using the forward primer 426-PGK1-F and the reverse primer 426-CYC1-R (Table S1). Then the amplified expression cassette was assembled into the linearized plasmid pRS426-*cdt-1* by the DNA assembler method (Shao et al., 2009) and the plasmid pRS426-*cdt-1-gh1-1* was gained. For plasmid pRS415-*cdt-1* and *pRS415-gh1-1*, the plasmid pRS415 was linearized by restriction enzyme XbaI and XmaI. The expression cassette of CDT gene and BGL gene with the *PGK1* promoter and the *CYC1* terminator were amplified by PCR with primer 415-*PGK1*-F and the reverse primer 415-*CYC1*-R (Table S1) and then were assembled into the linearized plasmid pRS415 by the DNA assembler method. The resulting plasmids were extracted and transformed into target strain by the LiAc/SSDNA/PEG procedure (Gietz et al., 1995) and transformants were selected on SC agar plates. The isolation of the plasmid from the evolved strain was performed by Yeast Plasmid Kit (Tiangen, Beijing, China) and amplified through *E. coli* transformation. Then the plasmid was extracted from *E. coli* and verified by sequencing.

### Evolution engineering

To improve the efficiency of the cellobiose utilization of strain SyBE001602, an evolution engineering approach was performed. The yeast strain SyBE001602 was cultured in YPC media till stationary phase, and then transferred to a new flask containing the same culture medium at an initial OD_600_ of 0.1. The serial subcultures were repeated 20 times for nearly 50 days until the specific growth rate of the yeast culture remained almost constant. After 20 times subculture, cells from shake flasks were streaked on YPC plates to isolate single colonies. Twenty colonies with the largest size were chosen and cultivated in YPC medium for 48 h, and then each colony was inoculated into 3 mL YPC medium in rubber-sealed test tubes at an initial OD_600_ of 0.1. All the test tubes were cultivated for 12 h (30°C and 150 rpm). The metabolites were analyzed regularly after cultivation and the colony with the highest ethanol production rate and cellobiose consumption rate was isolated and named SyBE001603.

### Batch fermentation

Yeast cells were cultivated in YPC medium to prepare inoculums for fermentation and cells were harvested by centrifugation (4000 rpm, 5 min) at mid-exponential phase. Flask fermentation experiments were performed in 50 mL YP medium containing 40 or 80 g/L cellobiose using 150 mL shaking flask (sealed by a rubber stopper with a syringe needle) at an initial OD_600_ of 1.0 under oxygen limited conditions. All the flask fermentations were performed in duplicates. Bioreactor fermentations were performed in 400 mL YP medium containing 80 g/L cellobiose in a 0.7-L bioreactor (Bailun, Shanghai, China) at 38°C with an agitation speed of 200 rpm under oxygen limited conditions. Initial cell densities were adjusted to OD_600_ ~ 1.

### Simultaneous saccharification and fermentation

The cellulase used for enzymatic hydrolysis was Celluclast (Sigma-Aldrich, St. Louis, MO, USA) with cellulase activity of approximately 80 FPU/mL and BGL activity of 20.1 CBU/mL (Ruffell et al., 2010). SSF experiments were conducted in 50 mL YP medium with 4% (w/v) Avicel (Sigma-Aldrich, St. Louis, MO, USA) with cellulase enzyme loadings of 10 FPU/g of glucan. The pH was adjusted to 4.8 by citrate buffer sterilized with 0.22 μm-filter. The YP medium was sterilized at 121°C for 20 min, and the Avicel was sterilized by UV light. The fermentation condition was the same with the flask fermentation method above.

### Analysis of substrates and fermentation products

Cell growth was monitored by optical density (OD) at 600 nm using a Model 722 grating spectrometer (Shanghai No. 3 Analysis Equipment Factory, Shanghai, China). Samples were taken periodically from the cultures and centrifuged at 10,000 rpm for 5 min to collect supernatant for metabolite analysis. Cellobiose, glucose and ethanol in the samples were determined by high performance liquid chromatography (HPLC) system consisting of a Waters 1515 pump (Waters, Milford, MA, USA), a Bio-Rad HPX-87H column (Bio-Rad, Hercules, CA, USA) and a Waters 2414 refractive index detector (Waters, Milford, MA, USA). The HPLC separation was operated at 65°C with 5 mM H_2_SO_4_ as mobile phase at a flow rate of 0.6 mL/min (Zha et al., 2012).

### Real-time PCR analysis

Yeast cells were cultivated under the same condition with flask fermentation at 30°C in YP medium with 40 g/L cellobiose. The cells were harvested at 12 h and washed by ice-cold phosphate-buffered saline. The total RNA was immediately extracted using TRIzol reagent (Invitrogen, Carlsbad, CA, USA) and reverse-transcribed into cDNA using RevertAid First Strand cDNA Synthesis Kit (Thermo Fisher Scientific, Waltham, MA, USA) following the manufacturer's instructions. Quantitative Real-time PCR was performed in the ABI Step one Plus Real-Time PCR system (Applied Biosystems, Foster City, CA, USA). The oligos used in the PCR were listed in Table S1. Three biological replicates were prepared for each gene. The data was normalized using *ACT1* as the internal standard and analyzed according to the 2^−Δ*ΔCT*^ method (Zha et al., 2014).

## Results

### Improved cellobiose utilization through evolution engineering

**Figure 1:**
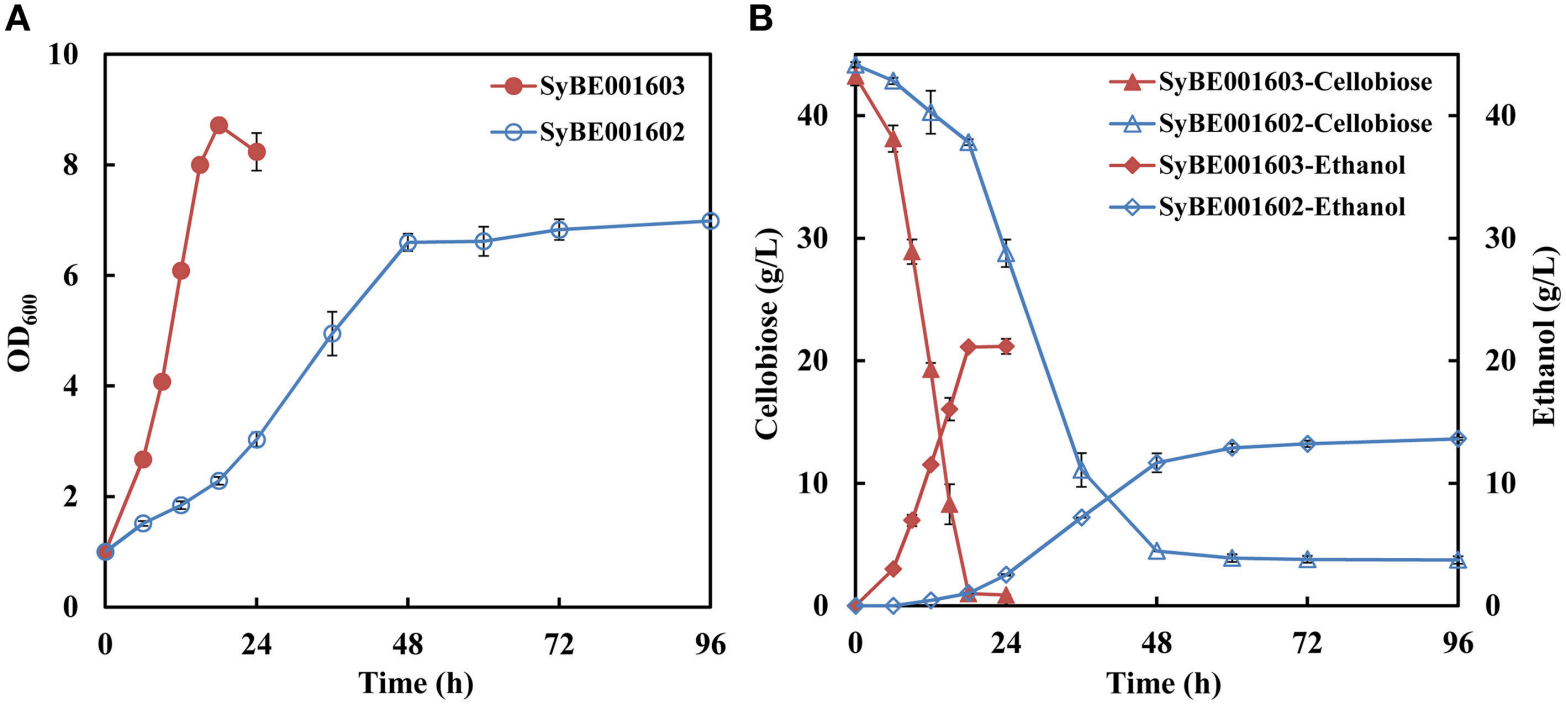
**Growth (A) and fermentation performance (B) of the parent strain SyBE001602 and evolved strain SyBE001603**.

A heterologous cellobiose utilization pathway consisting of CDT and BGL was introduced into industrial *S. cerevisiae* strain SyBE001601. The obtained strain, named SyBE001602, was able to produce ethanol when using cellobiose as substrate, but the growth rate and fermentation efficiency was low (Figure 1).

Evolution engineering was carried out to enhance the cellobiose utilization of the engineered strain. After the evolution a strain with rapid cellobiose utilization capability was obtained and named SyBE001603. A comparison of the evolved and parent strain was performed at 30°C with 40 g/L cellobiose. SyBE001603 consumed cellobiose quickly and completely in around 18 h, while SyBE001602 was not able to ferment all the cellobiose even after 96 h with 3.7 g/L cellobiose left at the end of fermentation (Figure 1B). In the fermentation with SyBE001602, the accumulation of cellodextrins was observed (Figure S1), which led to a low ethanol yield of 0.34 g ethanol/g cellobiose (Table 2). While almost no cellodextrins accumulation was observed in the fermentation with SyBE001603. The evolved strain SyBE001603 showed 2.8- and 4.9-fold higher cellobiose consumption rate and ethanol productivity than strain SyBE001602 (Table 2). The ethanol yield increased by 47% and reached 0.50 g ethanol/g cellobiose. According to the results, the cellobiose metabolism was significantly optimized by evolution engineering.

**Table 2:** **Comparison of the fermentation profiles of the parent strain SyBE001602 and evolved strain SyBE001603**.

	**Cellobiose consumption (g/L/h)**	**Ethanol productivity (g/L/h)**	**Yield (g ethanol/) g cellobiose)**
SyBE001602	0.83 ± 0.01	0.24 ± 0.02	0.34 ± 0.00
SyBE001603	2.35 ± 0.04^***^	1.17 ± 0.01^***^	0.50 ± 0.01^*^

Results are expressed as mean ± standard error of the mean. Significance levels of Students t-test:

* P < 0.05,

*** P < 0.001.

### Effect of temperature on cellobiose fermentation

To investigate the effect of temperature on cellobiose utilization, fermentation with SyBE001603 was performed respectively at 30, 34, 38, and 42°C. The strain exhibited the highest growth rate and cellobiose consumption rate at 34°C (Table 3). Cellobiose in the medium was completely consumed in 15 h and the average cellobiose consumption rate reached 2.61 g/L/h. Interestingly, the ethanol production rates and cellobiose consumption rates were almost the same at 30 and 38°C. Even at 42°C, 40 g/L cellobiose was consumed completely within 36 h, with an ethanol production rate of 0.61 g/L/h. The ethanol yield was around 0.49~0.50 g ethanol/g cellobiose at different temperature and no negative effect of high temperature on the ethanol yield was observed in this study. These results suggested that the strain SyBE001603 exhibited an advantage of thermotolerance in ethanol fermentation when using cellobiose as the sole carbon source.

**Table 3:** **Cellobiose consumption rate, ethanol productivity, and yield at temperature grads of 30, 34, 38, and 42°C**.

**Temperature (°C)**	**30**	**34**	**38**	**42**
Maximum specific growth rate (/h)	0.16 ± 0.01	0.18 ± 0.02	0.13 ± 0.02	0.06 ± 0.01^*^
Cellobiose consumption (g/L/h)	2.19 ± 0.00	2.61 ± 0.02^**^	2.19 ± 0.00	1.28 ± 0.00^***^
Ethanol productivity (g/L/h)	1.09 ± 0.03	1.27 ± 0.03^*^	1.05 ± 0.02	0.61 ± 0.03^**^
Yield (g ethanol/g cellobiose)	0.50 ± 0.03	0.50 ± 0.01	0.50 ± 0.01	0.49 ± 0.00

Results are expressed as mean ± standard error of the mean. Significance levels of Students t-test:

* P < 0.05,

** P < 0.01,

*** P < 0.001.

**Figure 2:**
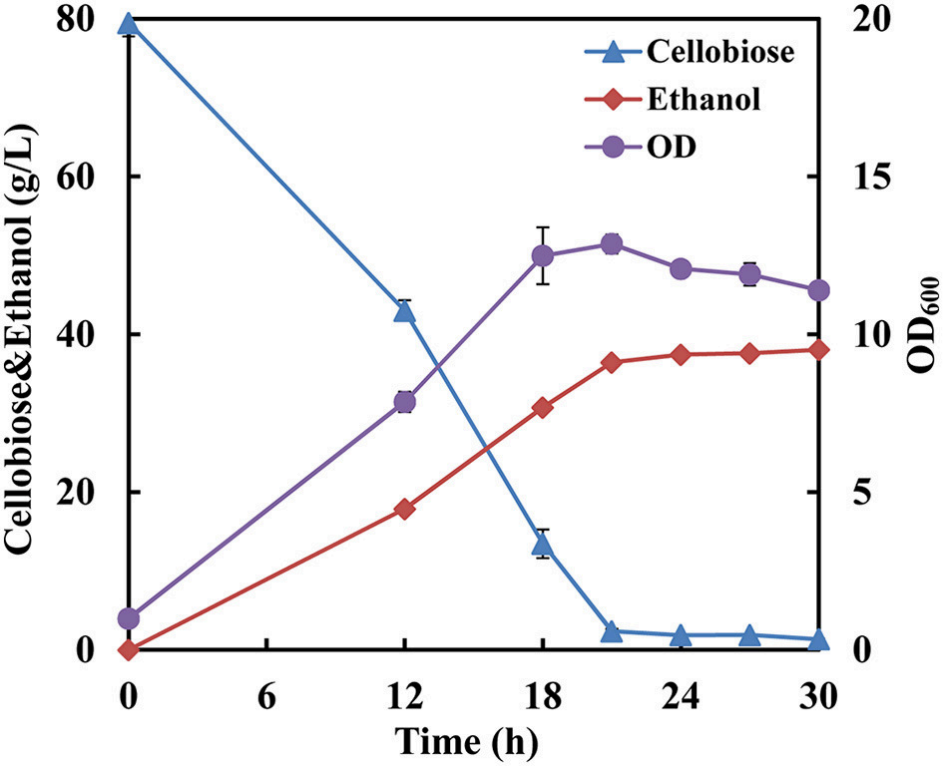
**Fermentation performance of the strain SyBE001603 with high cellobiose concentration**.

Since the strain SyBE001603 showed the highest cellobiose consumption rate at 34°C, fermentation with a high cellobiose concentration of 80 g/L was conducted at 34°C to further test its cellobiose utilization capability. The cellobiose consumption rate reached 3.67 g/L/h and almost all the cellobiose was consumed at 21 h (Figure 2). The ethanol production rate reached 1.73 g/L/h and the yield was 0.49 g ethanol/g cellobiose.

### Effect of pH on cellobiose fermentation

**Figure 3:**
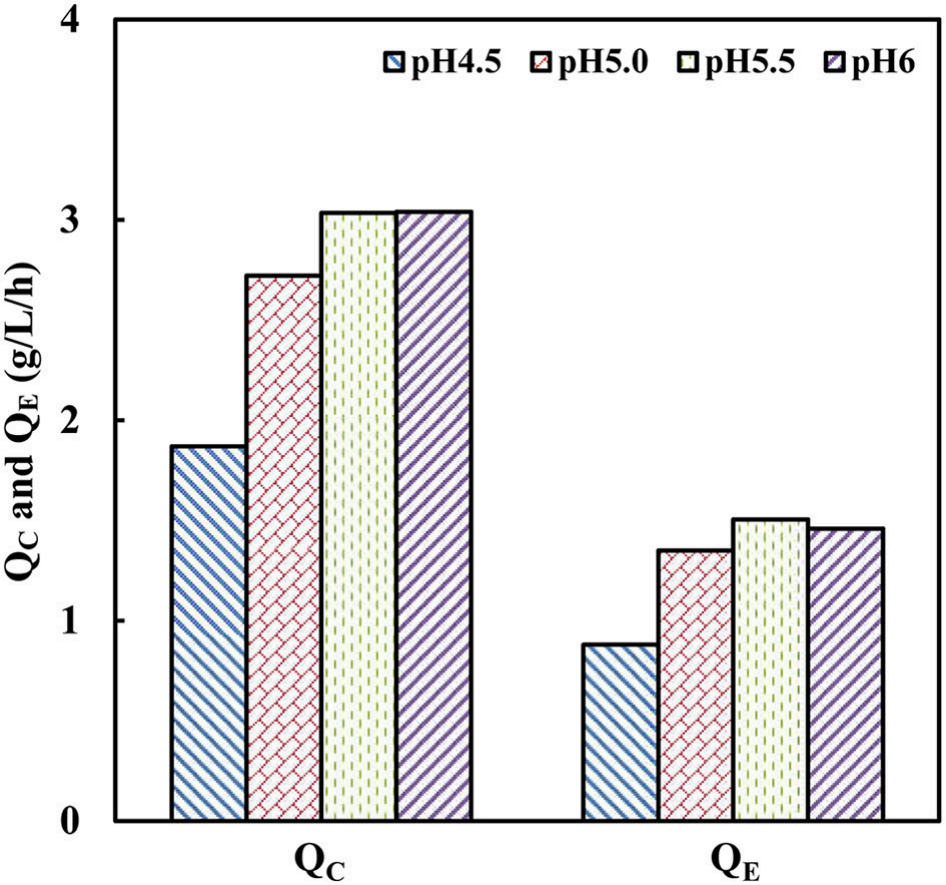
**Effects of pH on cellobiose fermentation by SyBE001603**. Q_C_, the average cellobiose consumption rate; Q_E_, the average ethanol production rate.

Generally, the optimum pH of cellulases is considered around 4.8 (Caminal et al., 1985). The industrial strain with tolerance toward low pH-values can facilitate efficient saccharification and minimize the risk of contamination (Olofsson et al., 2008). To investigate the effect of pH on cellobiose fermentation of strain SyBE001603, fermentation processes were performed at different pH of 4.5, 5.0, 5.5, and 6.0 in bioreactors at 38°C (Figure 3). The strain SyBE001603 presented the highest cellobiose consumption rate of 3.04 g/L/h at pH 5.5 and pH 6.0 and almost all the cellobiose was consumed within 24 h. The cellobiose consumption rate reached 2.72 g/L/h at pH 5.0, which was a bit lower than those at pH 5.5 and pH 6.0. At pH 4.5 the cellobiose was consumed completely within 42 h with a consumption rate of 1.87 g/L/h. As a result, strain SyBE001603 can be tolerant to a pH as low as 4.5 and has better fermentation performance at pH 5.0~6.0.

### Simultaneous saccharification and fermentation of cellulose

**Figure 4:**
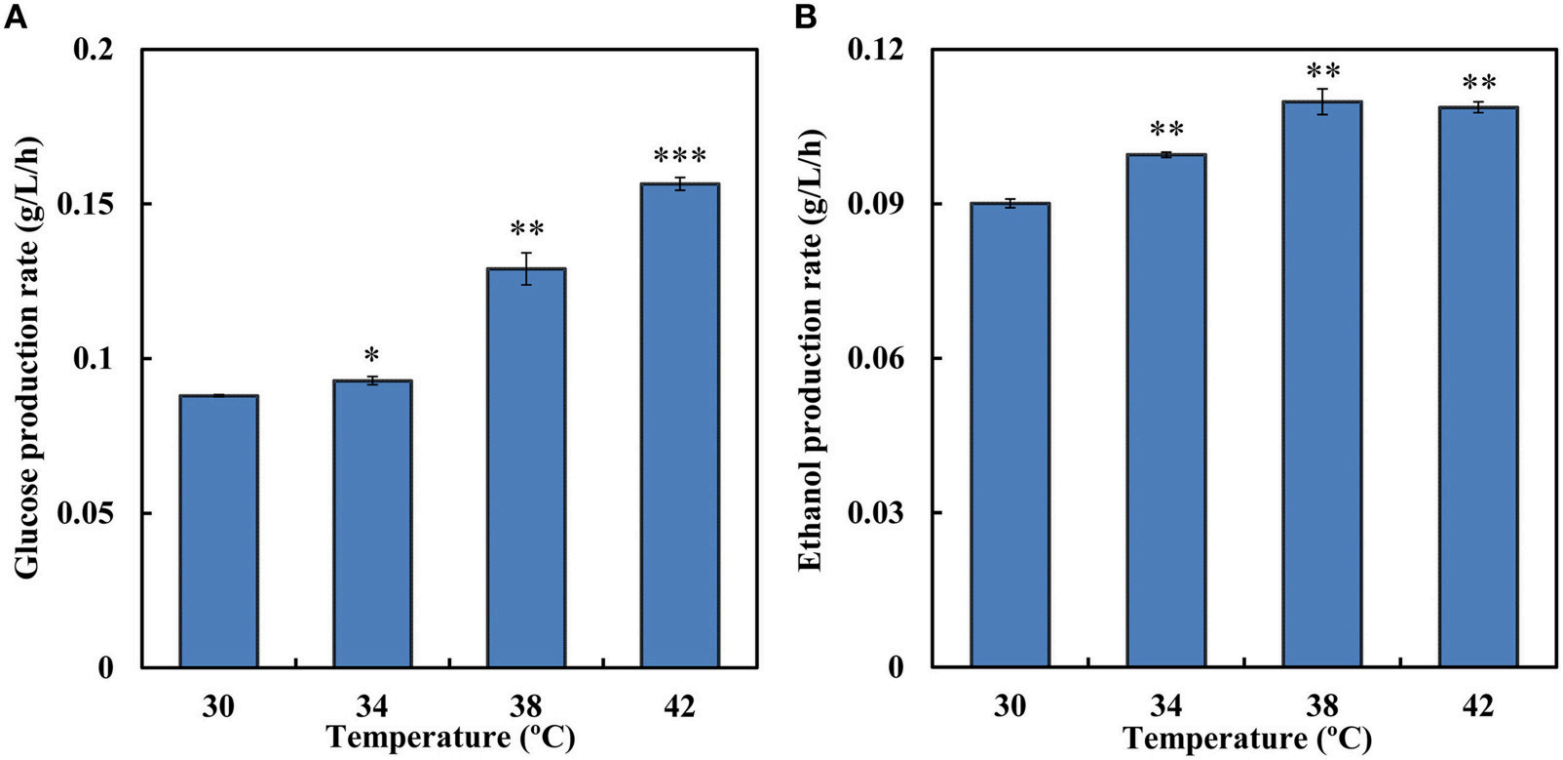
**(A)** The average equivalent glucose production rate in enzymatic hydrolysis at different temperature (0–144 h). **(B)** The average ethanol production rate during simultaneous saccharification and fermentation of SyBE001603 at different temperature (0–144 h). The equivalent glucose = the concentration of glucose contained in the released cellobiose + the concentration of glucose released. Results are expressed as mean ± standard error of the mean. Significance levels of Students *t*-test: ^*^P < 0.05, ^**^P < 0.01, ^***^P < 0.001.

During SSF, enzymatic hydrolysis and yeast fermentation were significantly affected by temperature. SSF and enzymatic hydrolysis were conducted at a temperature grads from 30 to 42°C to determine the optimum temperature. During the saccharification process 12.7 g/L and 13.4 g/L equivalent glucose were released at 30 and 34°C, respectively, while at 38°C the rate was 46.6% higher than that at 30°C (Figure 4A). Increases in temperature led to higher conversion rate of Avicel in enzymatic hydrolysis. Among the temperature grads, the optimal temperature for enzymatic hydrolysis was 42°C and 22.5 g/L equivalent glucose was released in total. However, the optimal temperature for strain SyBE001603 was 34°C (Table 3). In SSF process the ethanol production performance at higher temperature were better than at 30°C (Figure 4B). Till 144 h 15.8 g/L ethanol was produced at 38°C and 15.7 g/L ethanol was produced at 42°C, while only 12.9 g/L and 14.3 g/L ethanol was produced at 30°C and at 34°C during the fermentation, respectively. During the process the strain at 38°C showed better fermentation performance than other temperature, thus 38°C was suggested to be the optimum temperature for SyBE001603 in SSF, and the efficiency of enzymatic hydrolysis was obviously accelerated by SSF.

**Figure 5:**
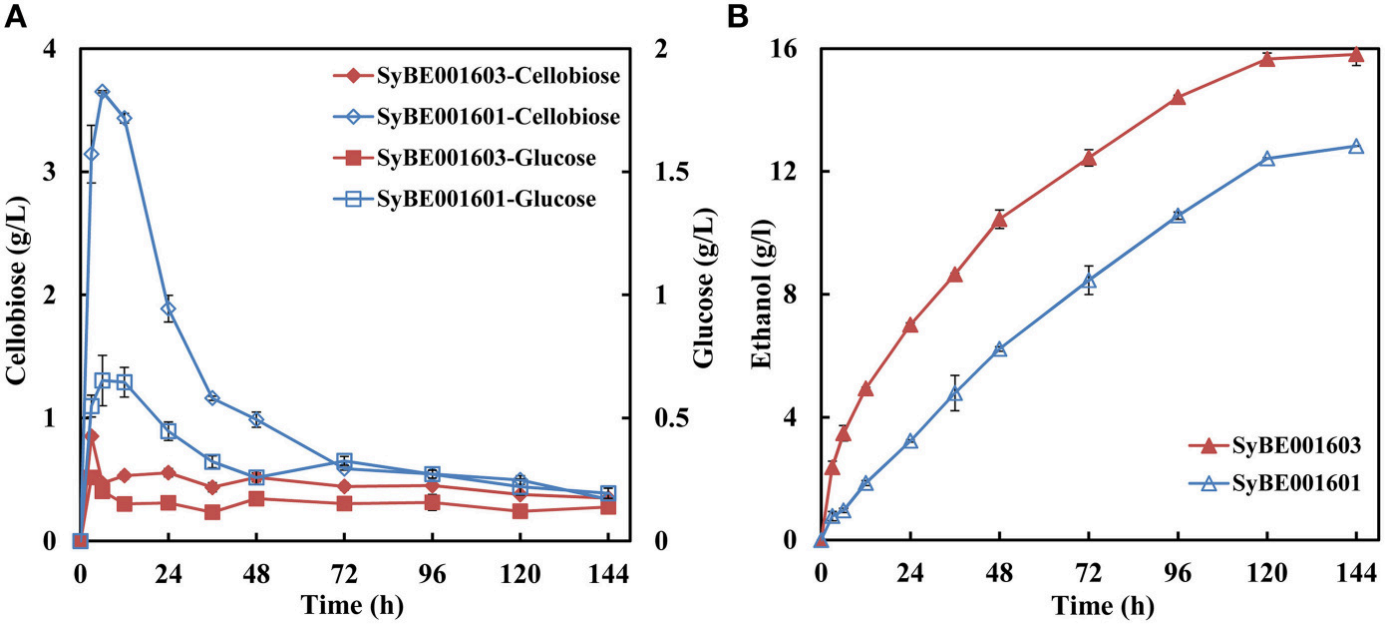
**(A)** Cellobiose and glucose concentrations and **(B)** Ethanol concentration during SSF using yeast strains SyBE001601 and SyBE001603 at 38°C.

SyBE001603 and its parent strain SyBE001601 were applied in SSF at 38°C. For SyBE001601 obvious cellobiose accumulation was observed at the initial stage and a peak value of 3.65 g/L appeared at 6 h (Figure 5A) while for SyBE001603 cellobiose was consumed and much less cellobiose (below 1 g/L) was accumulated. At 6 h when the cellobiose accumulation reached a peak value, the ethanol productivity of SyBE001603 was 2.6-fold higher than SyBE001601. The ethanol yield increased 23% from 0.34 to 0.42 g ethanol/g cellulose (Figure 5B). These results suggest that the inhibition of cellobiose and glucose on cellulase was significantly relieved by SSF with strain SyBE001603.

### Rational optimization of cellobiose utilization

To further explore the differences between the parent strain and evolved strain which led to higher ethanol fermentation efficiency, the relative transcription levels of the CDT gene and the BGL gene in strain SyBE001603 and SyBE001602 were measured by Real-time PCR. The CDT gene exhibited dramatically higher expression level in SyBE001603, which increased 5.74-fold compared to SyBE001602, and the BGL gene expression level increased 0.21-fold (Figure S2).

In order to investigate whether or not the increased expression levels of the CDT and BGL gene might influence the overall cellobiose utilization, five laboratory *S. cerevisiae* strains, BY4741, BY4742, YSG50, CEN.PK2-1C and L2612 were used as chassis strains to overexpress the CDT and BGL gene. The plasmid pRS426-*cdt-1-gh1-1* was transformed into the five parent strains, then the single-copy plasmids pRS415-*cdt-1* and pRS415-*gh1-1* were introduced into the resulting strains to overexpress the CDT and BGL gene, respectively. After overexpression of the CDT and BGL gene, the cellobiose utilization was improved obviously (Table 4). The improvement of cellobiose utilization was the most significant in BY4741 chassis. The cellobiose consumption rate of strain BY4741-Cell+CDT reached 0.98 g/L and increased 42% compared with its parent strain (0.69 g/L), and the cellobiose consumption rate of strain BY4741-Cell+BGL increased 23.5%. When overexpressing the gene in BY4742-Cell, the cellobiose consumption rate of strain BY4742-Cell+CDT and BY4742-Cell+BGL both increased 23%. For the other three chassis strains, the overexpression of either the CDT gene or BGL gene led to an improvement at around 13~17%.

**Table 4:** **Effects of overexpressed CDT or BGL gene on the fermentation performance in different chassis strains**.

	**Cellobiose consumption (g/L/h)**	**Ethanol productivity (g/L/h)**	**Yield (g ethanol/g cellobiose)**
BY4741-Cell	0.69 ± 0.01	0.29 ± 0.00	0.42 ± 0.00
BY4741-Cell+CDT	0.98 ± 0.01^***^	0.42 ± 0.01^**^	0.43 ± 0.00
BY4741-Cell+BGL	0.85 ± 0.00^**^	0.35 ± 0.01^*^	0.41 ± 0.01
BY4742-Cell	0.69 ± 0.00	0.29 ± 0.01	0.41 ± 0.01
BY4742-Cell+CDT	0.85 ± 0.00^**^	0.36 ± 0.01^*^	0.43 ± 0.01
BY4742-Cell+BGL	0.85 ± 0.00^***^	0.37 ± 0.00^**^	0.44 ± 0.00
YSG50-Cell	0.85 ± 0.00	0.38 ± 0.01	0.45 ± 0.02
YSG50-Cell+CDT	0.98 ± 0.00^***^	0.40 ± 0.01	0.46 ± 0.02
YSG50-Cell+BGL	0.98 ± 0.00^***^	0.43 ± 0.02^*^	0.45 ± 0.01
CEN.PK2-1C-Cell	0.77 ± 0.00	0.33 ± 0.01	0.43 ± 0.01
CEN.PK2-1C-Cell+CDT	0.86 ± 0.01^**^	0.36 ± 0.01^*^	0.42 ± 0.01
CEN.PK2-1C-Cell+BGL	0.87 ± 0.00^***^	0.37 ± 0.02^*^	0.43 ± 0.02
L2612-Cell	0.49 ± 0.00	0.20 ± 0.00	0.41 ± 0.01
L2612-Cell+CDT	0.58 ± 0.00^***^	0.23 ± 0.00^**^	0.40 ± 0.01
L2612-Cell+BGL	0.57 ± 0.01^**^	0.23 ± 0.00^**^	0.40 ± 0.01

Results are expressed as mean ± standard error of the mean. Significance levels of Students t-test:

* P < 0.05,

** P < 0.01,

*** P < 0.001.

The plasmid pRS426-*cdt-1-gh1-1* isolated from the evolved strain was sequenced to search any genetic mutations which may lead to improved cellobiose utilization but no mutations were found on the gene expression cassettes. The isolated plasmid was transformed into the chasis strain SyBE001601 and strain SyBE001604 was obtained to test the cellobiose fermentation property. As a result, compared to strain SyBE001602, SyBE001604 showed indistinctive cellobiose fermentation abilities (Figure S3).

## Discussion

In this study, a thermotolerant industrial *S. cerevisiae* was constructed to utilize cellobiose at a high temperature of 42°C. Cellulose (Avicel) can be converted efficiently into ethanol by this strain using SSF at 38°C with the yield of 0.42 g ethanol/g cellulose. The strain can be applied in the bioconversion of lignocellulosic materials to valuable bioproducts.

Cellobiose accumulation and its inhibition of cellulases is a daunting problem in the saccharification of pretreated biomass in the SSF (Zhao and Xia, 2009). BGL was supplemented besides cellulases during SSF in order to minimize cellobiose accumulation, which led to the increased cost of cellulolytic enzymes (Lee et al., 2013). In this study, efficient SSF can be achieved at a low cellulase loading of 10 FPU/g of glucan without the additional BGL by strain SyBE001603. The strategy has been reported by expressing the genes encoding cellobiose transportors discovered in *Neurospora crassa, Kluyveromyces lactis, Scheffersomyces stipites*, and *Penicillium chrysogenum* (Galazka et al., 2010; Sadie et al., 2011; Ha et al., 2013b; Bae et al., 2014). The cellobiose is transported into the cell and decomposed into glucose by the glucosidase *in vivo*, saving the cost for BGL and increasing the SSF efficiency. This method is quite different from the consolidated bioprocess (CBP), in which the constitutive enzymes of the cellulase mixture were expressed in a single cell or consortium to provide the cellulases for hydrolysis of pretreated biomass without addition of extra cellulases (Kim et al., 2013; Kricka et al., 2014). The CBP method can decrease the cost for cellulase cocktails but decreases the saccharification and fermentation efficiency as well since the yields and activities of the cellulases are not high enough (den Haan et al., 2015). The strategy used in this study is very efficient in cellulose hydrolysis and fermentation (Figure 5). Moreover, this alternative can help to bypass glucose repression, achieving simultaneous co-fermentation of cellobiose and xylose (Li S. J. et al., 2010; Ha et al., 2011; Kim et al., 2013).

In previous studies, most cellobiose-fermenting strains were constructed from laboratory strains, which show low tolerance to severe stimulus that are quite common in industrial applications such as a high concentration of sugars, high temperatures, and acidic environment. In this study, we used an industrial strain as chassis to express cellobiose pathway. This industrial strain is a commercial strain with robustness to high temperatures and other harsh stimulus, therefore has been applied in industrial ethanol production and brewing in China (Li et al., 2014; Liu Z. H. et al., 2014; Liu et al., 2016).

The ability of cellobiose utilization by the recombinant industrial strain SyBE001602 is very weak. Combinatorial transcriptional engineering and simultaneous engineering of multiple proteins have been applied to optimize the cellobiose pathway successfully (Du et al., 2012; Eriksen et al., 2013). Apart from rational engineering, evolution engineering, a random means, is also effective in improving the utilization ability of non-favored carbon sources (Dhaliwal et al., 2011; Hong et al., 2011; Zha et al., 2014). The evolved strain SyBE001603, obtained after 20 transfers in medium with cellobiose as the sole carbon source, showed significant improvement in cellobiose consumption and ethanol yield (Table 2). Cellodextrins accumulation in the medium was observed during the fermentation by SyBE001602 (Figure S1), which is probably ascribed to the transglycosylation activity of BGL and can reduce ethanol yield and ethanol productivity as well (Ha et al., 2011; Bae et al., 2014; Kim et al., 2014). However, almost no cellodextrins were accumulated during the fermentation of SyBE001603. Accordingly, the ethanol yield reached 0.49~0.50 g ethanol/g cellobiose, which is higher than reported in previous studies (Table S2). In addition, obvious cellobiose accumulation was also observed during the preliminary stage of the SSF by SyBE001602 with a peak value of 3.65 g/L whereas little cellobiose were accumulated for SyBE001603 (Figure 5). Therefore, the cellobiose accumulation is minimized by SyBE001603 during SSF and the inhibition of cellobiose on cellulases can be greatly relieved.

The improvement of cellobiose utilization may be related to the programmed expression of key genes in the cellobiose metabolism (Yuan and Zhao, 2013). In this study, the transcriptional level of CDT was upregulated significantly after evolution engineering (Figure S2). Increased expression of CDT might enhance the transportation of cellobiose into cell. Overexpression of the CDT gene in other five strains also demonstrates that sufficient expression of CDT can improve cellobiose consumption. As the first step of cellular metabolism, cellobiose transport is a rate-limiting step in the whole fermentation (Lian et al., 2014). The result showed that upregulation of BGL gene improved the cellobiose consumption and the ethanol productivity, indicating the important role of BGL in cellobiose metabolism. However, the effects of the two gene overexpressions on the improvement of cellobiose utilization were chassis-dependent, which might be due to the close relationship of cellobiose metabolism and cell-intrinsic metabolism. Besides the heterologous genes, the fermentation performance of *S. cerevisiae* was strongly influenced by the genetic background (Hector et al., 2011; Du et al., 2012). Moreover, since the CDT is reported to have cellodextrin transport abilities (Galazka et al., 2010), the enhanced expression of CDT gene might increase the cellobiose and cellodextrin transportation ability, and the cellodextrin accumulation can be relieved when the cellobiose transport capabilities increased (Ha et al., 2013b). Besides the absolute expression levels of the CDT and BGL genes, the BGL/CDT ratio are a key point to cellobiose utilization (Yuan and Zhao, 2013). After evolution engineering, the ratio of BGL/CDT decreased from 2.50 to 0.45, leading to a new balance of cellobiose metabolism with higher efficiency.

To explore the underlying mechanism for the evolution the plasmid pRS426-*cdt-1-gh1-1* from SyBE001603 was isolated and sequenced, but the sequencing result showed that no mutations occurred in the promoters and gene coding regions. Moreover, the transformant containing the plasmid extracted from SyBE001603 exhibited no significant improvement of cellobiose utilization compared to SyBE001602 (Figure S3). These discoveries indicate that the reprogramming of the transcription and translation process for CDT, BGL, and other relevant genes in the cellobiose metabolism may happen during the evolution. In an earlier study in our lab, the reshuffling of expression pattern for xylose metabolism was observed in the evolved strain without any changes to DNA sequences (Zha et al., 2014). Comparative analysis of the global transcripts and the genomic DNA of SyBE001602 and SyBE001603 will provide more valuable information for additional improvement of cellobiose metabolism.

Thermotolerance is another important issue for yeast strains in SSF (Lin and Tanaka, 2006). Compared with SHF, the efficiency of hydrolysis was lower in SSF because of low temperature compatible with yeast fermentation (Hasunuma and Kondo, 2012). Thereby, numerous attempts including genome shuffling and introducing thermo tolerant devices have been tried to obtain thermotolerant *S. cerevisiae* strains for SSF (Shi et al., 2009; Liu Y. et al., 2014). In the fermentation at 38°C, SyBE001603 showed a cellobiose consumption rate of 3.04 g/L/h and an ethanol production rate of 1.50 g/L/h, demonstrating the strong resistance of SyBE001603 to a high temperature. The cellobiose consumption rate and the ethanol production rate were 3.67 and 1.73 g/L/h at 34°C, respectively. To the best of our knowledge, SyBE001603 is the fastest cellobiose-utilizing *S. cerevisiae* strain at high temperatures ever reported (Table S2). In the future, the thermotolerance of SyBE001603 should be further improved to efficiently work in the SSF at a higher temperature.

The thermotolerant cellobiose-fermenting yeast developed in the present study provides an ideal strain for efficient utilization of cellulose using the SSF process. In the following studies, the strain can be engineered to utilize xylose in the biomass hydrolysates, and some genetic engineering for tolerance would strengthen the potential workhorse (Wang et al., 2015). Then the tolerant strain can be applied in the economic conversion of biomass to ethanol and other valuable chemicals.

## Conclusion

Efficient cellobiose utilization is the key to cellulose bioconversion during SSF. Evolution engineering was an efficient approach to improve the cellobiose utilization of the engineered yeast strain. The fast cellobiose utilization in industrial yeast enhanced ethanol production by reducing cellobiose accumulation during SSF at high temperature.

## Author contributions

MH designed the whole program, operated the experiment, analyzed the results and wrote the article. JZ contributed to the design of the program, and helped to construct the engineered strain. LH contributed to the fermentation work. YL gave help about writing and language. MS helped to construct the engineered strain. CZ gave help about writing and dicussion. BL helped about protocol design, experiment operation and writing. YY gave suggesion about protocol design, experiment operation and writing.

### Conflict of interest statement

The authors declare that the research was conducted in the absence of any commercial or financial relationships that could be construed as a potential conflict of interest.
