# Supplementary material for: Enhanced Bioconversion of Cellobiose by Industrial Saccharomyces cerevisiae Used for Cellulose Utilization
Source: Front Microbiol. 2016 Mar 3;7:241. doi: 10.3389/fmicb.2016.00241 (PMC4776165; doi:10.3389/fmicb.2016.00241)
Supplement: Supplementary file 1 [file Presentation1.pdf]

Supplementary Material

Enhanced bioconversion of cellobiose by industrial *Saccharomyces cerevisiae* used for cellulose utilization

Meng-Long Hu, Jian Zha, Lin-Wei He, Ya-Jin Lv, Ming-Hua Shen, Cheng Zhong, Bing-Zhi Li*, Ying-Jin Yuan

*Correspondence: Bing-Zhi Li, E-mail: bzli@tju.edu.cn

1.1 Supplementary Figures

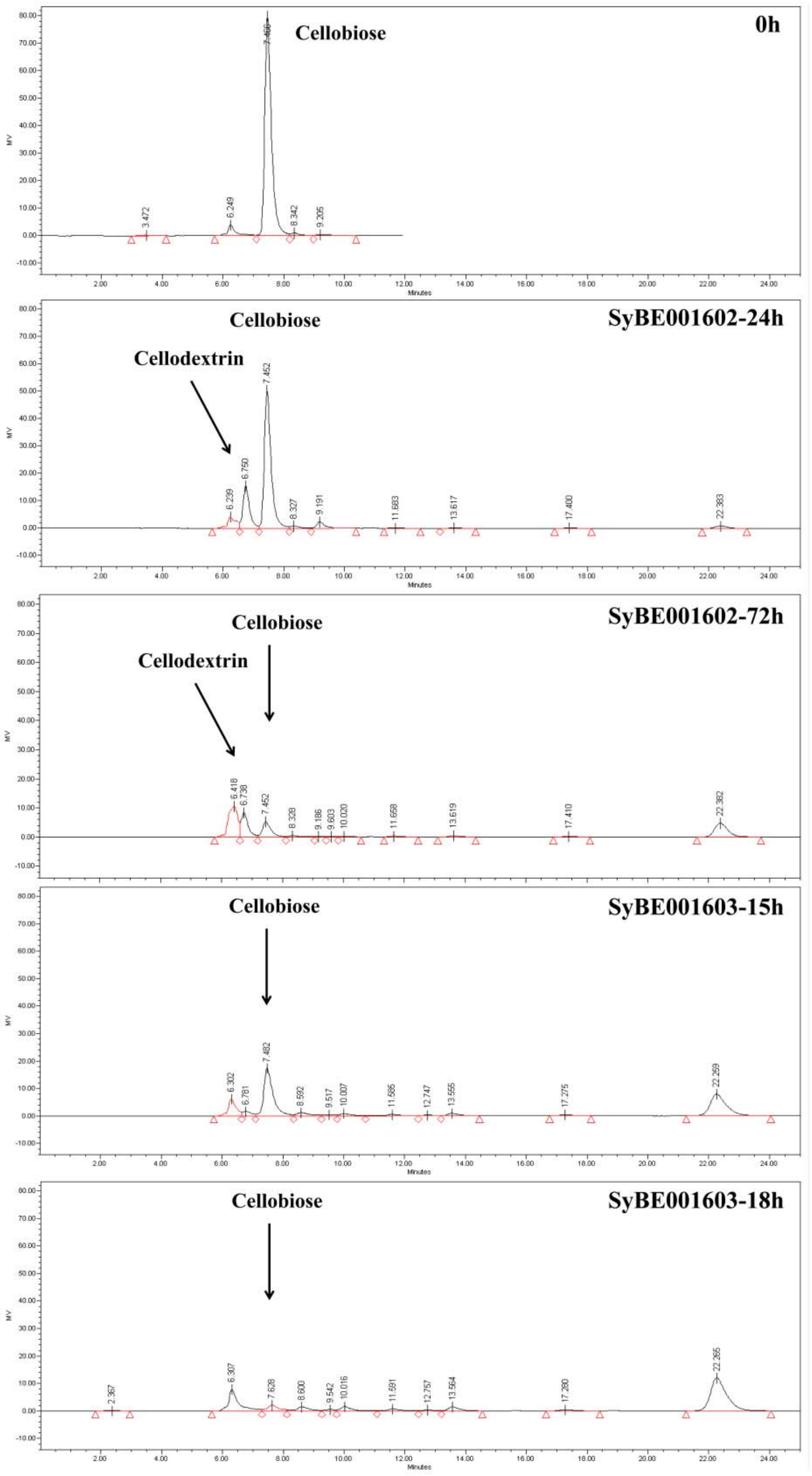

Figure S1. HPLC chromatograms showing cellodextrins accumulation during cellobiose

fermentation of strain SyBE001602 and SyBE001603.

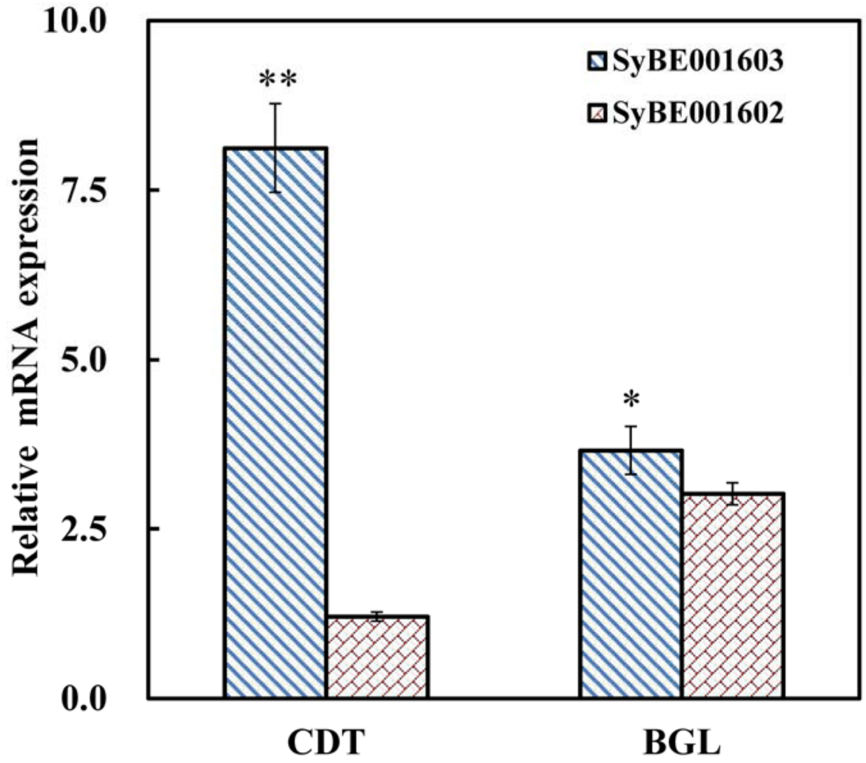

Figure S2. Transcription changes of the CDT gene and the BGL gene in the evolved and parent strain. Results are expressed as mean ± standard error of the mean. Significance levels of Students t-test: *P < 0.05, **P < 0.01~~, ***P < 0.001~~.

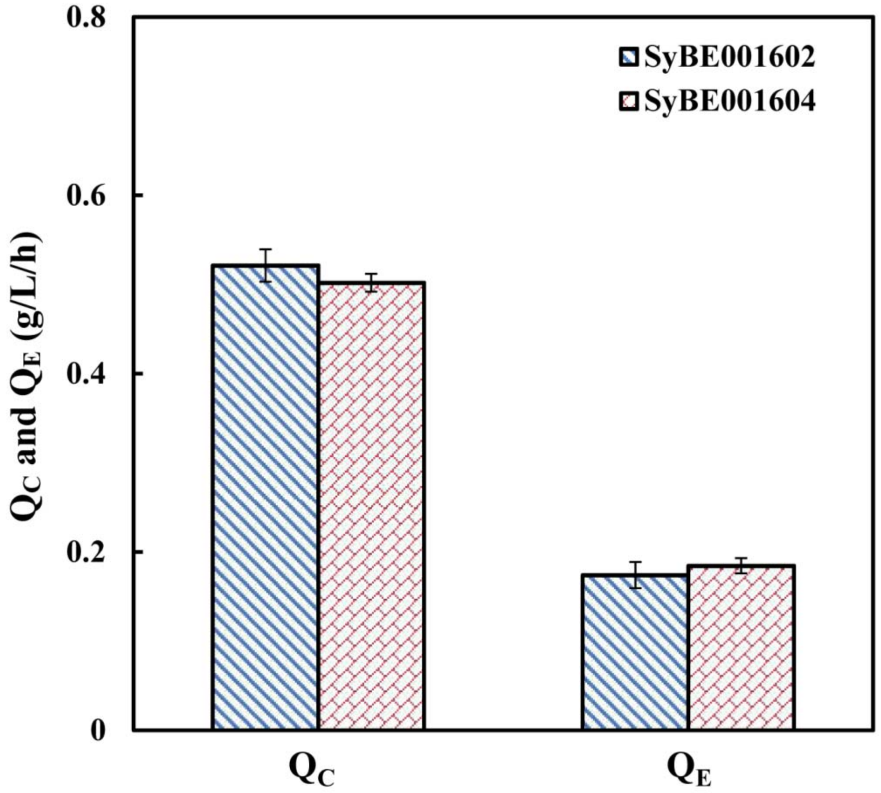

Figure S3.: ~~Comparision~~Comparison of the cellobiose utilization and ethanol production abilities between strain SyBE001602 and SyBE001604. Q_C: the cellobiose consumption rate in 72 h. Q_E: the ethanol production rate in 72 h.

1.2 Supplementary Tables

Table S1. Synthetic oligonucleotides used in this study.

Name	Sequence (5′to 3′)
426-PGK1-F	CGCCCGCTCCTTTCGCTTTCTTCCCTTCCTTTCTC GCCACGTTCGTGAGTAAGGAAAGAGTGAGGAA
426-CYC1-R	TCGGAACCCTAAAGGGAGCCCCCGATTTAGAGC TTGACGGGGAAAGGTACCGGCCGCAAA
415- PGK1-F	CGGTATCGATAAGCTTGATATCGAATTCCTGCAGCCCGGG GTGAGTAAGGAAAGAGTGAGGAAC
415-CYC1-R	ACAAAAGCTGGAGCTCCACCGCGGTGGCGGCCGCTCTAGA GGCCGCAAATTAAAGCC
ACT1-F	GAAATGCAAACCGCTGCTCA
ACT1-R	TACCGGCAGATTCCAAACCC
CDT-F	ATGAACGAGCAGGATGCCAA
CDT-R	GATCCCAGCCGACAAAGACA
BGL-F	GCCTACCGCTTCTCCATCTC
BGL-R	ATCGGGAAGATCCCAGTGGA

Table S2. Comparison of cellobiose fermentation ability of SyBE001603 with other engineered *S. cerevisiae* strains

Strain	Relevant genotype	Fermentation condition	Cellobiose consumption rate (g/L/h)	Ethanol production rate (g/L/h)	Ethanol yield (g/g)	Reference
SyBE001603	Evolved *cdt-1* and *gh1-1*	34°C, initial OD_{600}~1, oxygen-limited, YP medium with 80 g/L cellobiose	3.67	1.73	0.49	This study
SyBE001603	Evolved *cdt-1* and *gh1-1*	38°C, initial OD_{600}~1, oxygen-limited, YP medium with 80 g/L cellobiose	3.04	1.50	0.49	This study
D801-130	*cdt-1* and *gh1-1*	30°C, initial OD_{600}~1, oxygen-limited, YP medium with 40 g/L cellobiose	1.67	0.7	0.42	Ha et al. 2011
CTY-C59	*cdt-1* and *gh1-1*	30°C, initial OD_{600}~1, oxygen-limited, YP medium with 80 g/L cellobiose	2.18	0.74	0.39	Du et al. 2012
INV-C3	*cdt-1* and *gh1-1*	30°C, initial OD_{600}~1, oxygen-limited, YP medium with 80 g/L cellobiose	1.54	0.51	0.37	Du et al. 2012
Evolved CEN-HXT2.4-BGL	Evolved *HXT2.4* and *gh1-1*	30°C, initial OD_{600}~1, oxygen-limited, YP medium with 80 g/L cellobiose	2.08	0.88	0.43	Ha et al. 2013b
D452-HXT2.4(A291E)-BGL	Mutant *HXT2.4* and *gh1-1*	30°C, initial OD_{600}~1, oxygen-limited, YP medium with 80 g/L cellobiose	3.18	1.16	-	Ha et al. 2013b
R1	Evolved *cdt-1* and *gh1-1*	30°C, initial OD_{600}~0.2, oxygen-limited, YP medium with 80 g/L cellobiose	2.33	0.815	0.4236	Eriksen et al. 2013
R2	Evolved *cdt-1* and *gh1-1*	30°C, initial OD_{600}~0.2, oxygen-limited, YP medium with 80 g/L cellobiose	2.65	1.00	0.4363	Eriksen et al. 2013
Strain with CDT-1 (F213L) and SdCBP	Mutant *cdt-1 and SdCBP*	30°C, initial OD_{600}~1, oxygen-limited, YP medium with 80 g/L cellobiose	1.72	0.74	0.44	Ha et al. 2013a

D452-2	Mutant *cdt-1 and* SdCBP	30°C, initial OD_{600}~20, oxygen-limited, YP medium with 80 g/L cellobiose	3.6	1.5	-	Chomvong et al. 2014
SR8-a	Mutant *cdt-1 and* SdCBP	30°C, initial OD_{600}~20, oxygen-limited, YP medium with 80 g/L cellobiose	3.7	1.5	-	Chomvong et al. 2014
Pc ST/Tt BG	Pc ST and Tt BG	30°C, initial OD_{600}~1, oxygen-limited, YP medium with 40 g/L cellobiose	1.02	0.30	0.37	Bae et al. 2014 ~~JB~~
DCDT-1G	*cdt-1* and *gh1-1*	30°C, initial OD600~1, oxygen-limited, YP medium with 80 g/L cellobiose	-	0.53	0.39	Kim et al. 2014
DCDT-2G	*cdt-2* and *gh1-1*	30°C, initial OD_{600}~1, oxygen-limited, YP medium with 80 g/L cellobiose	-	0.11	0.19	Kim et al. 2014
